# Patient education in atopic dermatitis: a scoping review

**DOI:** 10.1186/s13223-023-00844-w

**Published:** 2023-10-13

**Authors:** Bethany Wilken, M. Zaman, Y. Asai

**Affiliations:** 1 Translational Institute of Medicine, Department of Medicine, Queen’s University, Kingston, ON Canada; 2 School of Medicine, Queen’s University, Kingston, ON Canada; 3 Division of Dermatology, Department of Medicine, Queen’s University, Kingston, ON Canada

**Keywords:** Atopic dermatitis, Eczema, Patient education, Therapeutic education, Self-management, Quality of life

## Abstract

**Background:**

Atopic dermatitis (AD) is a chronic inflammatory skin disease that affects children and adults. Poor treatment adherence in AD requires interventions to promote self-management; patient education in chronic diseases is key to self-management. Many international AD management guidelines published to date include a recommendation for educating patients as part of their treatment but there are no formal recommendations on how to deliver this knowledge.

**Main:**

We performed a scoping review to map the existing literature on patient education practices in AD and to highlight the clinical need for improved patient education in AD. The literature search was performed with the online databases MEDLINE, Embase, Grey Matters, ClinicalTrails.gov and the International Clinical Trials Registry Platform (ICTRP). The search strategy yielded 388 articles. Of the 388 articles screened, 16 studies met the eligibility criteria, and the quantitative data was summarized by narrative synthesis. The majority of studies were randomized controlled trials conducted in Europe, Asia and North America. Since 2002, there have been limited studies evaluating patient education in the treatment of AD. Frequent education methods used included group-based educational programs, educational pamphlets, individual consultations and online resources. Education was most commonly directed at caregivers and their children. Only one study compared the efficacy of different education methods. In all included studies, the heterogenous nature of outcome measures and study design limited the consistency of results. Despite the heterogeneity of studies, patient education was shown to improve quality of life (QoL), disease severity and psychological outcomes in AD patients.

**Conclusion:**

This scoping review highlights that patient education is effective in a variety of domains relevant to AD treatment. Further comparative studies and randomized trials with longer-term follow-up are needed to provide validated and consistent patient education recommendations for AD; these may depend on age and population.

**Supplementary Information:**

The online version contains supplementary material available at 10.1186/s13223-023-00844-w.

## Background

Atopic Dermatitis (AD), also known as eczema, is a chronic, relapsing-remitting, inflammatory skin disease that affects up to 20% of children [1]. AD has profound impacts on patient and caregiver quality of life (QoL). Pediatric and adult patients with AD experience pruritus, sleep impairment, social stigma, and negative mental health impacts [2, 3]. Since AD commonly affects children, the family impact of AD can be extensive, with parents reporting high stress and feelings of helplessness [4]. Cost of treatment adds to the burden of AD. Even if prescription treatments are covered, families are encouraged to make lifestyle changes such as purchase of specific clothing, emollients, soaps, detergents, and other items [5]. Direct financial costs of AD in the US are estimated to be $3.8 billion USD annually [6]. Direct costs, along with indirect costs such as time off due to doctor’s appointments, underline the need to optimize AD management.

There is no cure for AD, but it can be effectively controlled. Treatment adherence is poor; a study utilizing electronic monitoring found only 32% of patients followed their topical therapy in AD [7]. Factors contributing to poor outcomes include the complexity of treatment regimens, lack of knowledge, and corticosteroid phobia [8]. Nearly half of patients and caregivers cannot correctly identify the potency of commonly prescribed topical corticosteroids [9]. Incorrect application of topical therapy and poor adherence may result in poor clinical outcomes. Without proper management, psychosocial and financial difficulties are intensified, lowering the QoL for AD patients and families. If poor outcomes from inadequate adherence to treatment are misinterpreted as ineffectiveness of treatment, therapy may be inappropriately escalated.

Patient education is a key strategy to develop patient knowledge and foster skills required to help manage AD [10]. Patient education has been shown to improve QoL and treatment adherence in many chronic illnesses, including diabetes, asthma and cardiovascular disease [11]. Patient education has been established as one of four evidence-based cornerstones of asthma care [12] and the National Standards for Diabetes Self-Management Education and Support includes a validated, Diabetes Self-Management Education/Support education program that is widely used in clinical practice [13]. Patient education in the field of dermatology and specifically AD is less robustly developed. ‘Atopic schools’ established in some centres have proven effective in improving the management of AD but these programs differ in content, schedule, organization and evaluation [14]. Additionally, these ‘atopic schools’ are not feasible for healthcare centres with limited resources. The European, American, Japanese and Canadian guidelines for the management of AD recommend patient education programs as an adjunct to conventional therapy in AD [15–18]. Despite these recommendations there has been no evaluation of formalized and harmonized educational interventions.

Scoping reviews are conducted on a broad topic to examine the extent, range, and nature of research activity in a heterogenous topic to determine knowledge gaps and future directions [19].^,^ [20] This review was performed to provide a comprehensive, clinically useful summary of patient education in AD. The research questions this review aimed to address were:

1. What is known from the literature about patient education in AD and has it improved management of the disease?
2. What can be done to improve patient education for AD patients?

## Methods

The protocol was drafted using the Preferred Reporting Items for Systematic Reviews and Meta-analysis Protocols (PRISMA-ScR) [21].

Inclusion criteria: Publications were included if they were written in English and involved a method of patient education in AD. Studies with various outcome measures including clinical, psychological and quality of life measurements were included to consider different aspects of AD management. Pediatric and adult populations were incorporated. For the pediatric population, education aimed at parents and caregivers was also included. In order to investigate how well participants retained information from education and how it impacted their outlook on the disease, we included studies that had one outcome directly measured by patient responses including interviews, surveys or questionnaires. This means that education studies which solely included validated scoring methods in disease severity and QoL (most commonly the Dermatology Life Quality Index (DLQI)) were excluded. Quality of life and disease severity are unique to an individual and are multi-factorial. For the purposes of our review we felt the best way to understand patients experiences with AD educational tools was to extrapolate our findings from direct patient reports instead of indirect index scores.

Exclusion criteria: Publications were excluded if they focused on education in occupational or contact dermatoses or other skin diseases that are not AD. Opinion pieces and conference abstracts/posters were excluded.

### Databases and search strategy

Embase and MEDLINE were searched with no lower restriction on publication date until October 26, 2021. The results were exported to Covidence, a systematic review software. The search strategy was developed with an experienced librarian and refined through team discussion to include a search of Grey Matters and clinical trials registered with ClinicalTrials.gov and the International Clinical Trials Registry Platform (ICTRP).

Search strategy: ((atopic dermatitis.mp. or eczema.mp.)AND(patient education.mp. or therapeutic education.mp. or health education.mp. or consumer health information.mp. or action plan.mp.)AND(questionnaire.mp. or exp questionnaire/ or survey.mp. or patient interview.mp.)).

### Selection of articles

Two reviewers independently screened all abstracts from Embase and MEDLINE (B.W. and Y.A.), and searched Grey Matters, ClinicalTrials.gov and the ICTRP followed by full-text articles (where available) (B.W. and M.Z.). Disagreements were settled by a third-party reviewer if necessary (Y.A.).

### Data charting

The data-charting was developed by B.W. and 16 eligible studies were charted and analyzed. Type of education method, study design, number and age of study participants, outcome measures for each study and if the outcome measures significantly improved were charted (Table 1). Critical appraisal of individual sources of evidence included sample size, population, intervention and time at which outcome was measured.

**Table 1:** Characteristics of studies that focused on pediatric and caregiver AD education included in the scoping review

Reference	Follow-up	Study type	Treatment/control no.	Education Method	Outcome measurements	Outcome measure(s) with significant improvement after education.
Brown et al. 2018	1 month	RCT	11/26	Eczema action plan	QoL (IDLQI, CDLQI), provider knowledge and parent comfort with AD management (study designed questionnaire)	Provider knowledge
Cheong et al. 2018	Immediately after intervention & 4 weeks	Prospective, observational	N/A	Pharmacist counseling	AD knowledge, caregiver’s level of confidence and caregiver’s satisfaction of counseling	Post intervention: qualitative positive results in satisfaction and confidence 4 weeks: caregiver knowledge
Chinn et al. 2002	4 & 12 weeks	RCT	0.5-4yrs: 61/54 4-16yrs: 58/62	Single nurse consultation (30 min)	Family impact (FDI), QoL (IDLQI, CDLQI)	None
Liang et al. 2018	3 & 6 months	RCT	293/293	Education program (4, once-weekly group sessions)	Disease severity (SCORAD), QoL (IDLQI, CDLQI), family and patient knowledge (study designed questionnaire)	3 months: disease severity, IDLQI 6 months: disease severity, IDLQI, knowledge
LeBovidge et al. 2021	3 months	RCT	91/84	Caregiver handbook	AD symptoms (POEM), caregiver confidence in management (PASECI), disease severity (EASI), QoL (IDLQI, CDLQI), family impact (FDI)	Caregiver confidence
Muzzolon et al. 2021	2–5 months	Nonrandomized clinical trail	21/27	“Dermatitis Club” group program (two, 90- minute sessions)	Disease severity (SCORAD, EASI), QoL (CDLQI), family impact (FDI)	QoL, family impact, disease severity
Ohya et al. 2013	3 & 6 months	RCT	29/30	Booklet and parental education program (2- day program)	Primary: disease severity (SCOARD), symptom scores (pruritus and sleeplessness, score 0–10), family impact (FDI), corticosteroid anxiety scores (**), usage of corticosteroid (number of tubes and total weight of cream used)	3 months: symptom scores 6 months: corticosteroid anxiety, usage of corticosteroid, disease severity, symptom scores
Rolinck-Werninghause et al. 2015	2 weeks	Pre-post	N/A	Individual nurse consultations (30–45 min.)	Parental assessment of their self-confidence in care, child’s disease severity and its treatment	Parent self-confidence, severe symptoms
Ryu & Lee	6 weeks during intervention & 2 weeks after intervention complete	Pre-post with controls	32/77	School-based atopy care program (6 sessions, 40 min each)	Objective severity (SCORAD), subjective severity (SAS), sleep disturbance, QoL (CDLQI), parent knowledge (PK), parent self-efficacy (PE), compliance of parents (PC), QoL parent (AIS)	During intervention: knowledge, efficacy, subjective severity 2 weeks after intervention: none
Shi et al. 2013	Immediately after education	RCT	18/19	Eczema action plan	Patient/caregiver understanding regarding treatment regimen and understanding of eczema (study designed questionnaire).	Understanding treatment
Son & Lim 2014	2 weeks	Quasi-experimental	20/20	Web-based educational program	Disease severity (POEM), QoL (IDLQI), parental self-efficacy	QoL, self-efficacy
Staab et al. 2002	1 year	RCT	93/111	Parental training program (6, weekly group sessions)	Disease severity (SCORAD), QoL and treatment habits (study designed questionnaire), treatment costs and coping strategies (The Trier Scales of Coping).	Treatment habits, treatment costs, coping strategies

Abbreviations: POEM = Patient Oriented Eczema Measure, IDLQI = Infant’s Dermatology Life Quality Index, SCORAD = Scoring Atopic Dermatitis, EASI = Eczema Area and Severity Index, QoLIAD = Quality of Life Index for Atopic Dermatitis, CDLQI = Children’s Dermatology Life Quality Index, FDI = Family Dermatitis Index, STAI = State-Trait Anxiety Inventory, SAS = Subjective Atopic Dermatitis Survey, PK = Parent’s Knowledge on Atopic Dermatitis, PE = Parental Efficacy test, PC = Parent Compliance scale, AIS = Dermatitis Impact Scale, PASECI = Parental Self-Efficacy with Eczema Care Index

** “How much are you worrying about applying corticosteroids to your children’s skin?” (1 = no anxiety, 5 = very anxious)

## Results

**Fig. 1:**
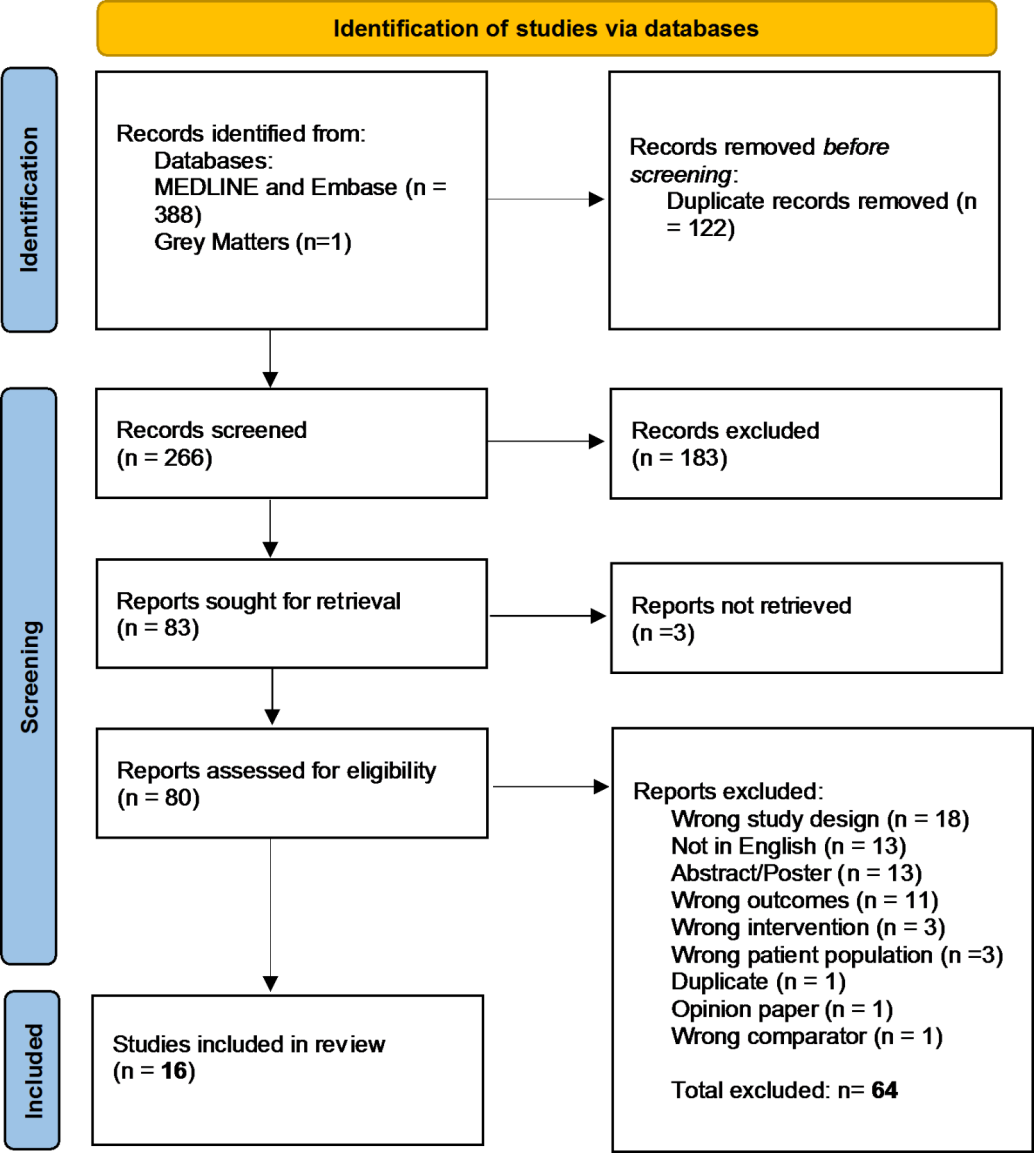
PRISMA-ScR flow diagram mapping the process used to include and exclude publications from the database searches. The databases searched were MEDLINE, Embase and Grey Matters

Of 388 results, 122 duplicates were removed and 266 were screened for title and abstract. Of these, 80 full-text publications were screened and 15 were included for data extraction. One additional study was identified by B.W. and M.Z. in the grey literature (Fig. 1). Active clinical trials registered with ClinicalTrials.gov and ICTRP were also searched on the topic and 4 were identified.

### Summary of selected studies

Sixteen studies examined associations between patient education and management of AD [22–37]. The majority were from the United States (n = 4), Korea (n = 2) and Germany (n = 3). There was one study each from the following countries: Belgium, England, Spain, China, Brazil, Singapore and Japan. Data from 4,541 participants was represented. The largest study included 1,628 participants [33], while the smallest evaluated 18 patients [35]. The largest study had the shortest follow-up time of 2 weeks [33]. Time to follow-up was broad, ranging from 2 weeks to 1 year. One study measured outcomes at three time points after education [23], 6 studies measured outcomes at two time points [25, 26, 29, 30, 32, 34] and 8 studies measured outcomes at one time point [22, 24, 27, 28, 31, 33, 35–37]. Of the included studies, the majority directed education towards in caregivers and children (n = 12) [24–26, 28, 29, 31–37]. Three studies reported on adults [22, 23, 27] and one study included all ages [30]. Most studies were randomized controlled trials (RCT; n = 11) [22–24, 26–30, 32, 35, 37] to examine the association between patient education and AD management. All studies evaluated multiple outcome measures; QoL (n = 9) [23, 24, 26, 27, 29, 31, 34, 36, 37] and disease severity (n = 10) [22, 23, 27–29, 31, 32, 34, 36, 37] were measured most frequently. Patient/caregiver knowledge or understanding (n = 5) [22, 25, 29, 34, 35], parental self-efficacy (n = 2) [34, 36] and depression/anxiety scores (n = 3) [27, 30, 32] were other outcome measurements.

### Patient education in AD: Children & Caregivers

Twelve studies educated children with AD and their caregivers (Table 1) [24–26, 28, 29, 31–37]. Most performed education in the presence of both child and caregiver while 3 studies targeted only parents/caregivers [25, 33, 37]. A variety of education methods were used including individual consultations with a healthcare professional [25, 26, 33], group based education sessions [29, 31, 32, 34, 37], eczema action plans [24, 35], a handbook [28] and the web-based education program that included self-assessment quizzes and written information about AD and its management (described in more detail below) [36].

### Individual consultations

One study investigated the efficacy of pharmacist counseling on caregiver’s knowledge, level of confidence in care and satisfaction of counseling [25]. The counseling was efficacious in improving caregiver knowledge [25]. The other outcome measures were measured qualitatively but showed positive results [25]. Interestingly, this study reported only 34% of caregivers had received previous explanations on AD [25], suggesting that over half of the AD caregivers received a prescription before a treatment explanation.

Two studies implemented single nurse educational consultations of similar times (30–45 min). [26, 33]. The content of the consultations in both studies were similar, focusing mainly on reinforcing care recommendations, demonstrating practical skills and establishing parent’s/child’s knowledge and understanding of eczema. Although the interventions are comparable, the studies used different outcome measures. Chinn et al. measured changes in family impact and QoL at two time points (4 & 12 weeks) following the nurse consultation and found no significant improvement [26], whereas Rolinck-Werninghaus et al. measured parental assessments of their self-confidence in care and the child’s subjective disease severity and found both of these measures were improved 2 weeks after the consultation.

### Group-based education

Group-based educational programs of varying lengths were used to educate AD children/caregivers in 5 studies [29, 31, 32, 34, 37]. Most were weekly sessions, occurring 4–6 times [29, 34, 37]. Group educational sessions were often interdisciplinary, led by nurse practitioners [29, 32], psychologists [37], pediatricians [37], dermatologists [29], elementary school teachers [34] and dieticians [37]. Two studies held group sessions with only caregivers present [32, 37]. The efficacy of these group programs was interpreted through measures of disease severity, QoL, knowledge, family impact, sleep disturbance, self-efficacy, treatment habits, treatment costs and coping strategies. Disease severity significantly improved after education in 60% (3/5) of the studies that measured this outcome [29, 31, 32], QoL improved in 50% (2/4 studies) [29, 31] and family impact improved in 33% (1/3 studies) [31]. Out of the other outcome measures, group education was shown to improve knowledge [29], self-efficacy [34], coping strategies [37] as well as treatment costs and habits [37]. Notably, one of these programs evaluated the effect of education on steroid phobia by measuring corticosteroid anxiety and corticosteroid use [32]. Significant improvements were seen in corticosteroid anxiety but there was no significant difference in the amount of corticosteroid used after education [32].

### Eczema action plan

Two American studies used written eczema action plans (EAPs) as an education tool [24, 35]. EAPs usually include written treatment instructions for maintenance therapy and mild-severe flares which can be supplemented with cartoons/pictures for younger ages [38]. Brown et al. used a generic EAP [24] while the other study tailored EAPs to each patient given their age, location, and disease severity [35]. The latter study found that the EAP improved the patient’s/caregiver’s understanding of treatment but not their understanding of the disease itself [35]. Uniquely, Brown et al. also considered the effect an EAP may have on the medical providers of AD patients. Although after one month of use their EAP was ineffective in improving patient comfort, understanding or QoL, it was effective in improving provider comfort and understanding [24]. It is worth noting that both studies had small sample sizes, with less than 20 participants in each intervention group [24, 35]. Sample EAPs from these two studies can be found in our supplemental information section.

### Online resource

Son & Lim developed a novel web-based education programme to overcome previous barriers in AD education [36]. The program was divided into two phases: Education I (Understanding of AD) and Education II (Management of AD), followed by a week of practice at home [36]. After each stage, online self-assessments were completed by participants. The results of these assessments showed that AD symptoms, QoL and self-efficacy were all improved 2 weeks after completion of the program [36].

### Caregiver handbook

LeBovidge et al. designed a handbook that was given to caregivers as a take-home tool to help manage the child’s AD [28]. The handbook focused on understanding, treating and managing AD and discussed topic such as improving sleep, dealing with emotional challenges and teaching kids how to be part of skincare [28]. The study found improvement in caregiver confidence in the management of AD symptoms, but did not see improvement in AD symtoms, disease severity, QoL or family impact [28].

### Patient education in AD: adults

Three studies educated adult patients with AD [22, 23, 27]. Similar to education in the AD pediatric population, outcome measures and education methods were diverse (Table 2).

**Table 2:** Characteristics of studies included in the scoping review that focused on adult AD education

Reference	Follow-up	Study type	Treatment/control no.	Education Method	Outcome measurements	Outcome measure(s) with significant improvement after education.
Armstrong et al. 2011	12 weeks	Comparative RCT	40/40	Pamphlet vs. online video	Disease severity (POEM), patient knowledge	Clinical severity, knowledge
Bostoen at al. 2012	3, 6 & 9 months	RCT	25/25	Group-based educational program (12 weeks)	Disease severity (SCOARAD, EASI, Skindex-29), QoL (QoLIAD)	3, 6 & 9 months: none
Heratizadeh et al. 2017	1 year	RCT	129/104	Group training (12 h)	Coping behaviour with itching (Juckreiz-Kognitions-Fragebogen questionnaire), social anxiety (Marburger Hautragebogen questionnaire), QoL, disease severity (SCORAD, Skindex-29)	Coping behaviour with itching, QoL, disease severity

Abbreviations: RCT = Randomized controlled trial, POEM = Patient Oriented Eczema Measure, SCORAD = Scoring Atopic Dermatitis, EASI = Eczema Area and Severity Index, QoLIAD = Quality of Life Index for Atopic Dermatitis

### Group-based education

In adults with AD, two studies facilitated extensive education through group sessions [23, 27]. Parallel to group education with children and caregivers, the adult group education programs emphasized interdisciplinary care, with instruction by dermatologists [23, 27], psychologists [23, 27], dieticians [23, 27], a dermatological nurse [23] and a sports, yoga and mindfulness coach [23]. Bostoen et al. studied the effectiveness of 2 h, twice weekly sessions concentrated on the patient’s skin disease, education on a healthy lifestyle and application of stress-reducing techniques [23]. After the program, disease severity, depression severity and QoL were assessed at 3, 6 & 9 months which revealed that there were no notable differences in scores between education participants and those who did not attend the group sessions [23]. In contrast, Heratizadeh et al. demonstrated that 12 h of a group training program significantly improved coping behavior with itching, QoL and disease severity [27].

### Pamphlet vs. online video

The only comparative study in this review, Armstrong et al., investigated the effectiveness of an AD education pamphlet versus an online educational video for adults [22]. The video and pamphlet had information pertaining to clinical manifestations of AD, contributing environmental factors, bathing and hand-washing techniques, moisturizer vehicles, and common treatment modalities [22]. Despite both mechanisms of knowledge delivery containing identical information, the online video showed significantly greater decreases in disease severity and greater increases in AD knowledge [22].

### Patient education in AD: all ages

One study looked at educating AD patients of all ages with the same tool [30]. Using an information booklet, investigators sought to determine how education impacts the emotional status of AD patients [30]. This study was the largest RCT in the review, with 564 participants in the intervention group and 683 controls. Emotional status was measured by levels of anxiety through the State-Trait Anxiety Inventory (STAI). The booklet containing information on important everyday patient-oriented aspects of AD only significantly improved STAI scores in ages 9-15yrs [30]. No other age groups showed benefits 3 or 6 months after receiving the booklet.

## Discussion

We identified 16 interventional studies addressing patient education in AD across various settings and populations. We found that educating patients and caregivers in AD is currently a diverse practice, with limited cohesiveness on the methods used and the goals of education. Our findings indicate that patient education can improve several aspects of the complex disease, including QoL, clinical severity and key factors of self-management [22, 24, 25, 27–37].

Despite these promising results, some studies showed mixed association between patient-education and AD improvements, while two studies showed no significant improvements in multiple domains [23, 26]. The first of these found no significant improvements in disease severity, depression severity or QoL between controls and an adult group-based education program for patients with mixed phenotypes of psoriasis and AD. Regarding immune mechanisms and treatment, psoriasis and AD are fundamentally different diseases [39, 40]. Assuming that the education program focused on overlapping aspects of both diseases, overlooking self-management knowledge and skills specific for AD could have limited its effectiveness in this disease population. Additionally, the study had a small sample size of 25 participants, with only 10 participants in the intervention group having AD [23]. This likely decreased statistical power to the point where significant differences were undetectable.

The second study employed a single nurse consultation for caregivers and their children aged 0.5-16yrs which was unsuccessful in improving family impact and QoL scores [26]. The investigators address several limitations that may have influenced outcomes including the type of medical practice, the characteristics of the patients and their parents, the numbers recruited, the follow-up period and the choice of outcome measures [26]. Unlike many other patient education studies which took place in secondary care settings, this study occurred in primary care with general practitioners. Participants were not selected based on disease severity subgroups and QoL scores on average, were lower than scores reported from secondary care populations [26, 41, 42], implying that the AD population in primary care may have milder disease activity. If baseline measures were low, it may have been more difficult to detect a meaningful change in this group.

### Strengths and Limitations

A strength of this review is its incorporation of data from clinical trial registries and the grey literature, which is frequently excluded from traditional databases. Although selection bias is always possible, the inclusion of studies with negative results supports this search strategy. Restriction to studies published in English could lead to bias, although it is reassuring that the included articles come from a variety of countries.

One possible limitation of this scoping review is that it could be missing important papers due to the search criteria. As previously stated, studies were only included if they used patient-reported outcomes that are not related to disease severity. The main goals of patient education are to improve the patient’s knowledge and to empower them in order to facilitate self-management of the disease. Studies should try to quantify how effective the education tool was in achieving these goals using patient-reported outcomes such as knowledge scores, self-efficacy, self-confidence and psychological measures. Some AD patient education studies which only include QoL or disease severity scores [43–46] risk capturing the variability of external influences more so than the direct effects of education.

### Future directions

#### Children and caregivers

Caregivers are important targets of education as the onset of AD occurs in 45% of children during the first 6 months of life, 60% during the first year of life and 85% before the age of 5 years [47]. Most studies targeted caregivers and their children. Few studies examined one education method for all ages. Targeting education to all ages of patients may not be ideal as learning styles and content may differ with phases of psychosocial development and disease trajectories [48]. There have been no studies to establish the time at which education should be directed toward children rather than parents. Considering parents spend 2 to 3 h per day caring for a child with AD, [49] defining the ideal age to directly educate the patient is essential in enabling self-management early on. Educating children in AD may pose challenges such as poor literacy and learning difficulties [50, 51]. For these reasons, education programs should be individually tailored to patients’ educational backgrounds. Alternatively, patient education guidelines suggest material should be written at sixth-grade or lower reading level, preferably including pictures and illustrations [52].

#### Adults

Although AD is commonly viewed as a pediatric disease, studies suggest adult AD is more frequent than previously recognized [53, 54]. Only three studies directed education at adults [22, 23, 27], suggesting education for adults with AD is extremely underdeveloped. AD can have a large impact on QoL in adults as occupational and psychosexual difficulties are well described [55] and a subset of adults have severe AD that is challenging to manage [56]. Education in the form of a training manual significantly improved coping behaviour, QoL and disease severity in adults with AD [27]. This supports the idea that patient education could help overcome challenges unique to adult AD and thus, requires more attention. However, being mindful that many adults with disease persistent from childhood experience heightened frustration and distrust stemming from prolonged exposure to the healthcare system [57], this attention and the aims of education should be distinct from the pediatric population.

#### Optimal delivery method

The available literature consists of a variety of study designs and education evaluations which makes direct comparison between studies impossible. Future RCT are needed to identify specific modalities (e.g., health care provider consultations vs. self-directed education) that would be more effective than others in achieving improved patient and caregiver confidence and competence in managing AD. The most effective methods are still being investigated to help develop standard education models. Current clinical trials in the United Kingdom, the United States and Japan are investigating pediatric AD education through methods of pharmacist led education, an educational video vs. handout and an allergy educator, respectively [58–60]. One Swiss clinical trial is evaluating the efficacy of a twice weekly educational session in adults [61].

When developing standard models, it is important to consider educating patients efficiently with available resources. A position paper from the International Eczema Council stated that education can improve QoL and patient satisfaction, but lack of funding and excessive bureaucracy limits its widespread implementation [62]. Many education interventions involved teaching by highly specialized and multidisciplinary professionals, [23, 29, 31, 34, 37] who are not widely accessible to AD patients nor feasible to hire in smaller health centers with limited resources. The COVID-19 Pandemic and the ageing “baby boomer” population has placed further pressure on providers caring for patients with AD [63, 64], highlighting the need for education methods that do not require the extensive training of professionals. It is worth devising and evaluating novel patient education tools that make the cost:benefit ratio of patient education more favourable.

### Outcome measures

Evaluation of patient education is a complex but essential process, but is currently fragmented, as evidenced by the variable outcome measures across studies. To encompass the key features of patient education, assessment should include a biomedical outcome, QoL scores and specific psychological scores [10]. The ultimate goal of patient education is to provide patients knowledge to make autonomous decisions, [65] yet there are currently no known validated AD knowledge questionnaires. Efforts should be focused on developing patient-reported outcome tools capable of assessing acquired skills and knowledge. When evaluating education by surveys and questionnaires, patients and caregivers may interpret the nature of the questions differently depending on age, individual characteristics and backgrounds. For instance, DLQI scores have shown to be significantly higher for non-white patients compared to white patients with the same disease severity [66]. Evidence is needed to distinguish how variation in interpretation contributes to the observed differential effectiveness in patient education studies.

### Follow-up

Considering the persistent, chronic nature of AD, appropriate follow-up in future studies is important to measure the efficacy of educational programs. The follow-up time in the educational studies included in this review ranged from two weeks to one year. Heratizadeh et al. 2017 demonstrated the long-term effectiveness of a training manual, as significant improvements in coping behaviour, QoL and disease severity were observed one year after the education intervention [27]. A group educational program for children also showed long term benefits, improving disease severity, QoL and knowledge after 3 and 6 months [29]. However, two weeks after educational intervention, no significant improvements were seen in disease severity in two studies [34, 36]. It has been suggested that educational interventions in AD should be followed up at least 1–3 months [67]. Prior to one month, disease severity may not be an accurate measurement as treated flare-ups can take several weeks to clear.

It may also be beneficial to perform follow-ups during different seasons. It is well known that AD flares are associated with seasonal changes, with increased temperatures predicting increased likelihood of AD office visits [68]. AD flares are commonly induced by viral illnesses [69] thereby, worsened disease severity in the winter may coincide with the increased prevalence of influenza and acute upper respiratory tract viral illnesses [70, 71]. Low temperatures in the winter can also cause AD by the drying of skin and spring season is associated with pollens, the main group of allergens that some patients feel worsen their AD symptoms [72]. If follow-ups are conducted in a different season than when the educational intervention took place, it could confound outcomes in disease severity.

## Conclusion

This scoping review summarized the available data relating to the use of patient education in the treatment of AD to help guide future management. Compared to other chronic diseases, the lack of official recommendations for patient education in AD treatment is substantial. The significant improvements patient education facilitated in disease management support an increased effort to improve the quality of AD education provided in the clinical setting. Accurately measuring the effectiveness of patient education remains a challenge. The mixed associations in some studies highlights the need for higher quality, comparative research to identify optimal methods for AD education administration. Future studies with longer follow-up are needed to further prove patient education’s fundamental role in AD management.

### Electronic supplementary material

Below is the link to the electronic supplementary material.

Supplementary Material 1

## Data Availability

All research analysed in this review is available through the online databases MEDLINE, Embase, Grey Matters, ClinicalTrials.gov or the International Clinical Trials Registry Platform repositories.
