# Supplementary material for: Patient education in atopic dermatitis: a scoping review
Source: Allergy Asthma Clin Immunol. 2023 Oct 13;19:89. doi: 10.1186/s13223-023-00844-w (PMC10576377; doi:10.1186/s13223-023-00844-w)
Supplement: Supplementary file 1 — Supplementary Material 1 [file 13223_2023_844_MOESM1_ESM.docx]

| **Citation** | **Resource/Link to resource** |
| --- | --- |
| Brown, J., Weitz, N. W., Liang, A., Stockwell, M. S., & Friedman, S. (2018). Does an eczema action plan improve atopic dermatitis? A single-site randomized controlled trial. Clinical pediatrics, 57(14), 1624-1629. | The Eczema Action Plan can be found in Figure 1 in Brown et al's., full text article. We have also included a copy at the end of this document |
| Lebovidge, J., Rea, C., Timmons, K., Delano, S., Greco, K., DeFreitas, F., ... & Schneider, L. (2021). A Randomized Controlled Trial of an Educational Handbook for Caregivers of Children with Atopic Dermatitis. Journal of Allergy and Clinical Immunology, 147(2), AB28. | Lebovidge reports on a patient handbook that was created, this handbook is not provided in the article or supplemental information. The methods of the paper states that handbook content was drawn from the following resources: **1)** Barbarot S, Bernier C, Deleuran M, et al. Therapeutic patient education in children with atopic dermatitis: position paper on objectives and recommendations. Pediatr Dermatol. 2013;30(2):199-206, **2)** Barbarot S, Stalder JF. Therapeutic patient education in atopic eczema. Br J Dermatol. 2014;170(Suppl 1):44-48., **3)** Schneider L, Tilles S, Lio P, et al. Atopic dermatitis: a practice parameter update 2012. J Allergy Clin Immunol. 2013;131(2):295–299.e291-227, **4)** Eichenfield LF, Tom WL, Berger TG, et al. Guidelines of care for the management of atopic dermatitis: section 2. Management and treatment of atopic dermatitis with topical therapies. J Am Acad Dermatol. 2014;71(1):116-132, **5)** Sidbury R, Tom WL, Bergman JN, et al. Guidelines of care for the management of atopic dermatitis: section 4. Prevention of disease. flares and use of adjunctive therapies and approaches. J Am Acad Dermatol. 2014;71(6):1218-1233, **6)** Klinnert MD, Booster G, Copeland M, et al. Role of behavioral health in management of pediatric atopic dermatitis. Ann Allergy Asthma Immunol. 2018;120(1):42-48.e48, **7)** Chida Y, Steptoe A, Hirakawa N, Sudo N, Kubo C. The effects of psychological intervention on atopic dermatitis. A systematic review and meta-analysis. Int Arch Allergy Immunol. 2007;144(1):1-9. |
| Shi, V. Y., Nanda, S., Lee, K., Armstrong, A. W., & Lio, P. A. (2013). Improving patient education with an eczema action plan: a randomized controlled trial. *JAMA dermatology*, *149*(4), 481-483. | An example of Shi et al's Eczema Action Plan can be found in appendix 2 in their full text article. We have also included a copy at the end of this document |

**Brown et al., Eczema Action Plan**


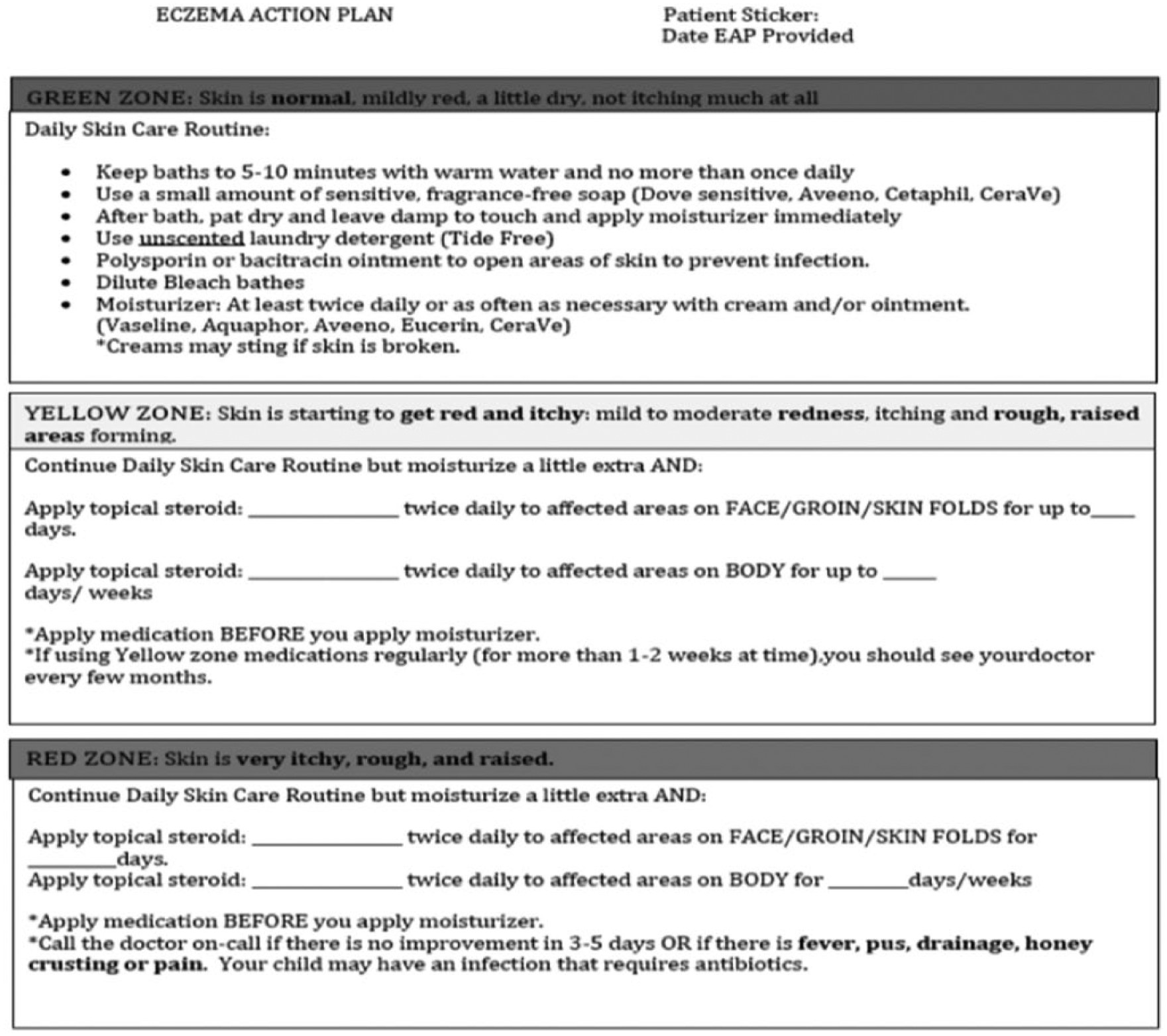


**Shi et al., 2013**

**
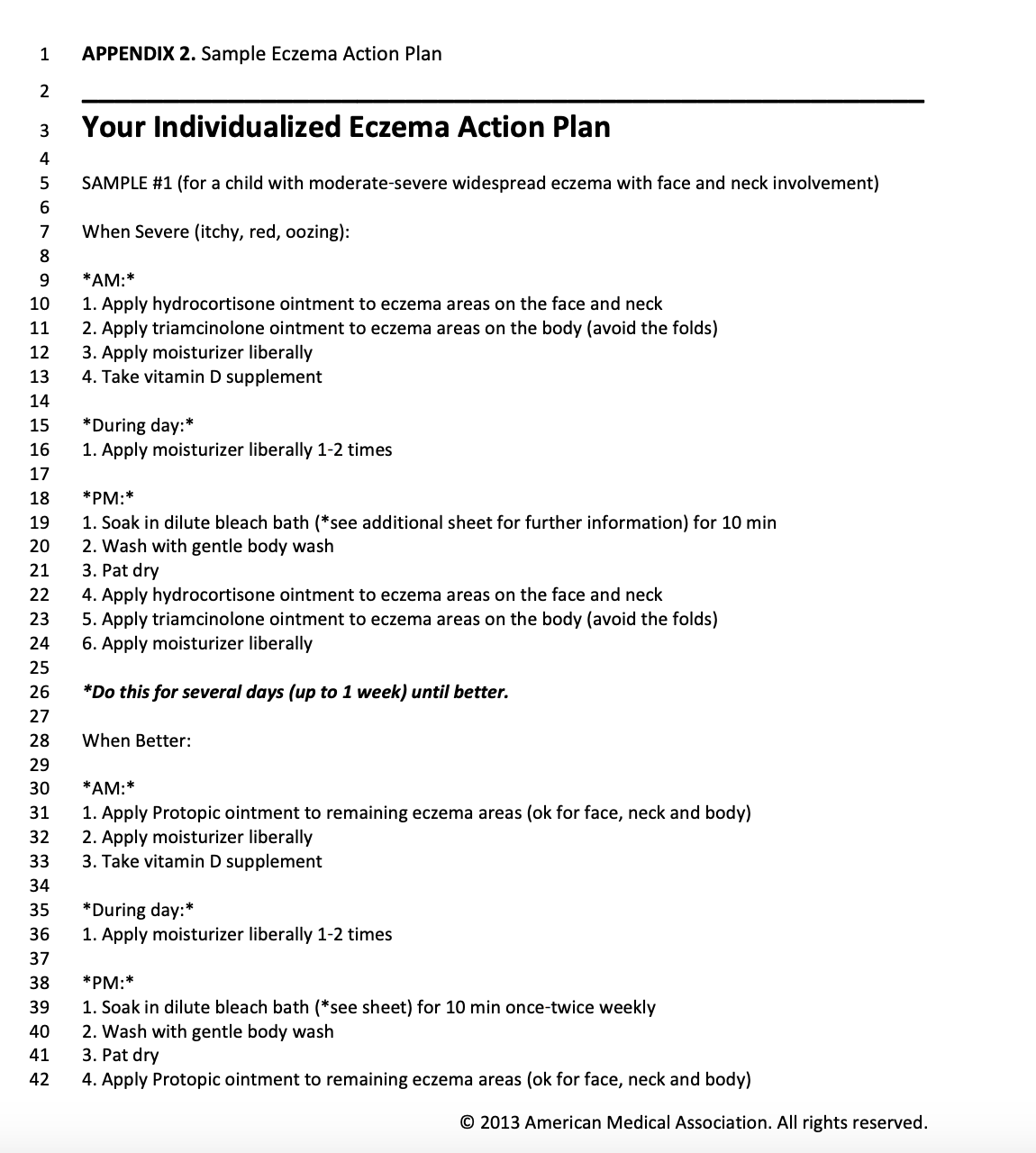
**
